# Molecular Characterization of Equine *Staphylococcus aureus* Isolates Exhibiting Reduced Oxacillin Susceptibility

**DOI:** 10.3390/toxins11090535

**Published:** 2019-09-13

**Authors:** Anissa D. Scholtzek, Dennis Hanke, Birgit Walther, Inga Eichhorn, Sabita D. Stöckle, Katja-Sophia Klein, Heidrun Gehlen, Antina Lübke-Becker, Stefan Schwarz, Andrea T. Feßler

**Affiliations:** 1 Institute of Microbiology and Epizootics, Centre for Infection Medicine, Department of Veterinary Medicine, Freie Universität Berlin, 14163 Berlin, Germany; anissa.scholtzek@fu-berlin.de (A.D.S.); dennis.hanke@fu-berlin.de (D.H.); inga.eichhorn@fu-berlin.de (I.E.); antina.luebke-becker@fu-berlin.de (A.L.-B.); stefan.schwarz@fu-berlin.de (S.S.); 2 Advanced Light and Electron Microscopy (ZBS-4), Robert Koch Institute, 13353 Berlin, Germany; waltherb@rki.de; 3 Equine Clinic: Surgery and Radiology, Freie Universität Berlin, 14163 Berlin, Germany; sabita.d.stoeckle@fu-berlin.de (S.D.S.); katjasophia.klein@gmail.com (K.-S.K.); heidrun.gehlen@fu-berlin.de (H.G.)

**Keywords:** borderline oxacillin resistance, BORSA, susceptibility testing, MSSA, whole genome sequencing, *blaZ*

## Abstract

The detection of borderline oxacillin-resistant *Staphylococcus aureus* (BORSA) represents a challenge to both, veterinary and human laboratories. Between 2015 and 2017, 19 equine *S. aureus* with elevated minimal inhibitory concentrations for oxacillin were detected in routine diagnostics. The aim of this study was to characterize these isolates to identify factors possibly associated with the BORSA phenotype. All *S. aureus* were subjected to antimicrobial susceptibility testing and whole genome sequencing (WGS). A quantifiable β-lactamase activity assay was performed for a representative subset of 13 isolates. The WGS data analysis of the 19 BORSA isolates identified two different genomic lineages, sequence type (ST) 1 and ST1660. The core genome multilocus sequence typing (cgMLST) revealed a close relatedness of all isolates belonging to either ST1 or ST1660. The WGS analysis identified the resistance genes *aadD*, *dfrG*, *tet*(L), and/or *blaZ* and *aacA-aphD.* Phenotypic resistance to penicillins, aminoglycosides, tetracyclines, fluoroquinolones and sulfamethoxazole/trimethoprim was observed in the respective isolates. For the penicillin-binding proteins 1–4, amino acid substitutions were predicted using WGS data. Since neither transglycosylase nor transpeptidase domains were affected, these alterations might not explain the BORSA phenotype. Moreover, β-lactamase activity was found to be associated with an inducible *blaZ* gene. Lineage-specific differences regarding the expression profiles were noted.

## 1. Introduction

*Staphylococcus aureus* can be a harmless commensal residing on the skin and mucosal surfaces of healthy animals and humans [1], but it can also cause a broad spectrum of diseases, ranging from relatively mild skin infections to life-threatening pneumonia, sepsis and endocarditis [2,3]. The infections caused by antimicrobial-resistant staphylococci are a major threat in human and veterinary medicine, since treatment options are often limited [3,4,5]. A broad range of different staphylococcal species has been detected in horses, including *S. aureus* and *Staphylococcus delphini* as coagulase-positive species, but also a variety of coagulase-negative staphylococci [6,7,8,9,10]. Several studies on equine *S. aureus* and, in particular, equine methicillin-resistant *S. aureus* (MRSA) have been performed in recent years [11,12,13,14,15].

Besides MRSA, also borderline oxacillin-resistant *S. aureus* (BORSA) gained particular attention [16]. Commonly, BORSA were initially defined by their reduced susceptibility to oxacillin with minimal inhibitory concentrations (MICs) of 1–2 mg/L, but some BORSA strains can also exhibit oxacillin MICs of 4–16 mg/L [16]. These isolates lack an additional penicillin-binding protein (PBP), encoded by either *mecA* or *mecC*, which are located on mobile genetic elements, the Staphylococcal Cassette Chromosome *mec* (SCC*mec*) in MRSA [1,16] or *mecB* carried on a plasmid, as recently described [17].

*S. aureus* isolates are assigned to clonal complexes (CC) based on their multi locus sequence types (STs). Since 2005, livestock-associated MRSA (LA-MRSA), in Europe attributed to CC398, became a public health issue, colonizing animals and humans. In addition, LA-MRSA were described for human cases of wound infection, deep abscess, cellulitis, necrotizing fasciitis and bacteremia [18,19]. In Europe, CC398 with *spa* type t011, but also other *spa* types such as t034 and t6867, is the most common clonal complex among MRSA in horse clinics [6,11,13,20,21,22,23,24,25,26,27]. However, several studies also observed a low number of isolates (MRSA and methicillin-susceptible *S. aureus* (MSSA)) attributed to CC1 with *spa* type t127 [6,11,21,25,26]. This clonal complex is known to be also associated with infections in humans, causing the majority of staphylococcal bacteremia in Denmark [28]. In other countries, such as England [29], Spain [30], Norway [31], Turkey [32] and Italy [33], CC1 is detected at lower frequencies. Other studies focusing on equine clinics found isolates with *spa* types t549 [6,25,26] and t3034 [21,26], which belong to ST1660. This ST has not been assigned to a clonal complex so far.

Previous studies showed an increase of both, MRSA and BORSA in equine clinics [20,21]. Moreover, there has been evidence that, once resistant staphylococcal isolates enter a human or veterinary clinic [11,34,35], the number of nosocomial infections—in particular wound infections [34,35]—increases. Nosocomial settings provide a selective pressure to bacterial pathogens since antimicrobial agents and also biocides are widely used [36,37]. While horse clinics have been identified as “hot spots” for pathogens accumulating antibiotic resistance in the recent past, little is known about frequency and dimension of biocide resistance. Consequently, more research is needed in this particular field and biocide susceptibility of field isolates should be monitored. However, studies determining MICs for biocides often lack an approved method and are therefore difficult to compare [37].

The aim of this study was to thoroughly characterize *S. aureus* of equine origin with elevated oxacillin MICs ranging from 0.5 mg/L to ≥4 mg/L via VITEK2 while lacking known methicillin resistance genes, such as *mecA* and *mecC*.

## 2. Results

### 2.1. Molecular Typing of Equine BORSA Isolates

**Figure 1:**
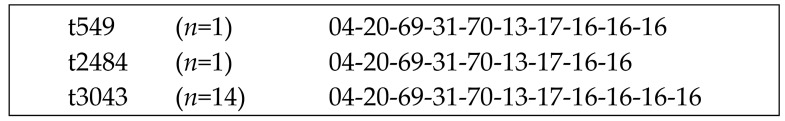
Comparison of the *spa* types, obtained for the ST1660 isolates.

The investigation of the whole genome sequences (WGSs) of the 19 isolates revealed two multi locus sequence (MLS) types, ST1 (*n* = 3, allelic profile: 1-1-1-1-1-1-1) and ST1660 (*n* = 16, allelic profile: 6-79-6-47-89-70-61). All ST1 isolates had *spa* type t127 (repeats: 07-23-21-16-34-33-13), whereas the ST1660 isolates displayed three different *spa* types, namely t549 (*n* = 1, IMT41899), t2484 (*n* = 1, IMT39637) and t3043 (*n* = 14). A comparison of these *spa* types revealed only differences in the number of the terminal repeats (Figure 1):

### 2.2. Phylogenetic Analysis

**Figure 2:**
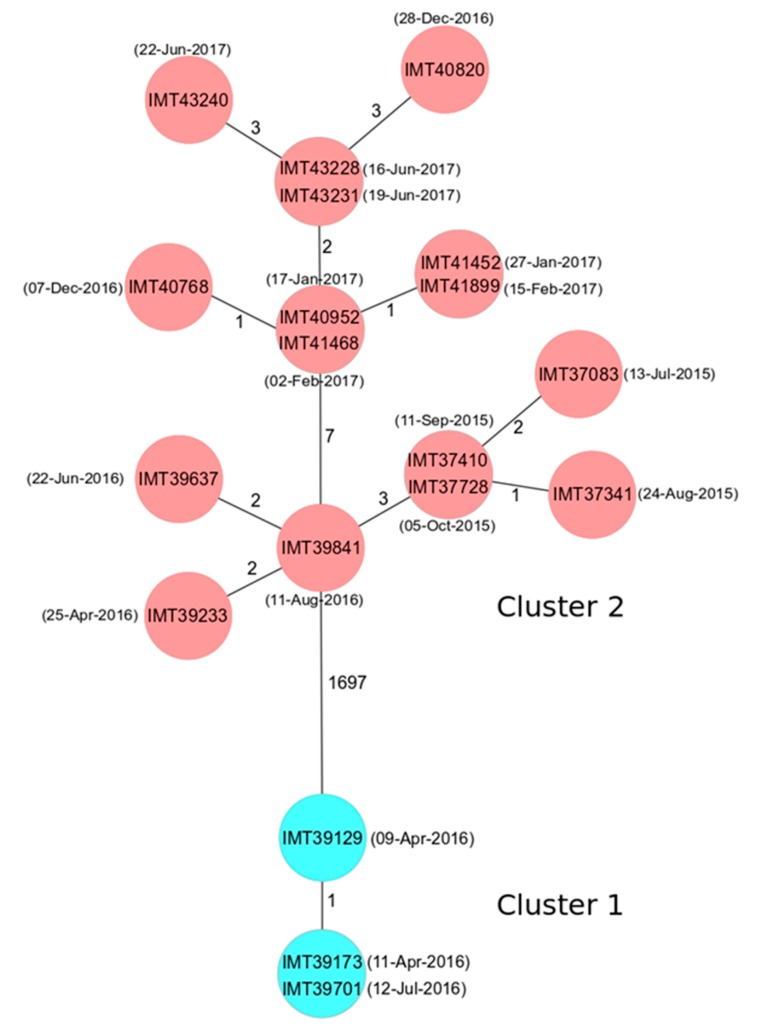
The minimum spanning tree showing the clonal relationship of 19 borderline oxacillin-resistant *S. aureus* (BORSA) isolates based on a core genome multilocus sequence typing (cgMLST) analysis including 1744 genes using the SeqSphere+ software. Each circle represents an allelic profile and the connecting lines display the number of different alleles between the distinct profiles. The individual isolate IDs are shown within the circles, the collection date is given in brackets, while the sequence types (ST) types are indicated by color: ST1 in light blue, ST1660 in red.

The core genome multilocus sequence typing (cgMLST) analysis revealed 14 different allelic profiles and two different clusters for the 19 BORSA isolates (Figure 2). Cluster 1 represented the ST1 sequences and consisted of two closely related allelic profiles differing only in one target gene. Cluster 2 included the ST1660 isolates assigned to 12 distinct, but also closely related allelic profiles differing in 0 to 7 target genes only. The phylogenetic relationship of the two clusters was considerably distant, mirrored by the differences in 1697 of 1744 alleles included in the comparison.

### 2.3. Virulence Factors of the 19 Equine BORSA Isolates

The molecular characterization based on WGSs revealed that all isolates harbored genes encoding the *S. aureus* bicomponent gamma-hemolysins (HlgA, HlgB and HlgC), alpha-hemolysin (Hla) and delta-hemolysin (Hld). Moreover, all ST1 isolates were positive for a β-hemolysin (Hlb) converting phage, which functionally inactivated the *hlb* gene (Table 1).

All isolates harbored a similar set of genes associated with adherence, including *ebpS* (encoding the elastin binding protein) and *efb* (encoding the extracellular fibrinogen binding protein). All isolates, except isolates IMT37341, IMT39841 and IMT41899, were positive for the fibronectin binding protein gene *fnbA*. Isolate IMT41899 was also negative for the gene *fnbB*. All isolates harbored the intercellular adhesion gene cluster (*ica*), which is associated with biofilm formation.

The ST1660 isolates carried genes for staphylococcal enterotoxins (SE) and SE-like toxins associated with the *egc* enterotoxin gene cluster, namely *sei*, *selm*, *seln*, and *selo*. Moreover, pseudogene *ϕent2* was present in all but one [IMT39841] and *selq* was present among all ST1660 isolates, while the ST1 isolates harbored the enterotoxin gene *seh*. All isolates harbored the leukotoxin-encoding genes *lukD* and *lukE*. All isolates were positive for the bacteriophage Saeq1 (acc. no. LT671578) harboring genes (*lukP* and *lukQ*) which code for a further leukocidin (Table 1). For the staphylococcal complement inhibitor (Scin)—also encoded on this phage—96% amino acid (aa) identity compared to the reference, bacteriophage Saeq1, was observed. All isolates were negative for the genes encoding the toxic shock syndrome toxin 1 (*tst*) and the Panton-Valentin leucocidin (PVL) genes *lukF-PV* and *lukS-PV*.

### 2.4. Antimicrobial Resistance Properties

Antimicrobial susceptibility testing was performed via broth micro- and macrodilution according to the Clinical and Laboratory Standards Institute (CLSI) recommendations [38,39]. The MIC value distribution and their classification into the categories susceptible, intermediate (if available) or resistant is displayed in Table 2. Penicillin MIC values from 2 to ≥64 mg/L were determined for the 19 isolates reported. Hence, all isolates were classified as resistant [38,39] and harbored the β-lactamase gene *blaZ*.

Broth microdilution revealed comparatively low oxacillin MICs of 0.25 or 0.5 mg/L for the ST1 isolates, and, as expected, the WGSs lacked the methicillin resistance genes *mecA*, *mecB* or *mecC*. Resistance to gentamicin and kanamycin, tetracyclines, and the combination sulfamethoxazole/trimethoprim was observed. The respective aminoglycoside resistance genes *aacA-aphD* and *aadD*, the tetracycline resistance gene *tet*(L) as well as the trimethoprim resistance gene *dfrG* were detected in the WGSs. The enrofloxacin MICs of 0.5 or 1 mg/L classified the respective isolates as resistant and the analysis of the quinolone resistance determining regions (QRDR) of GyrA/GyrB and GrlA/GrlB [40,41] revealed that the isolates displayed the same aa alteration Ser80Tyr in GrlA compared to a wildtype sequence (acc. no. D67075.1).

The ST1660 isolates had comparably higher MICs for oxacillin of 1 or 2 mg/L. Resistance to gentamicin and kanamycin was found in all isolates, which corresponded well to the presence of the *aacA-aphD* gene. The neomycin MIC values of 0.5 or 1 mg/L were lower than those obtained for the ST1 isolates with MICs of 8 or 16 mg/L, which might be caused by the absence of the *aadD* gene, which confers kanamycin/neomycin resistance. A single isolate was classified as intermediate to erythromycin. In comparison to the ST1 isolates, the ST1660 isolates displayed lower enrofloxacin MICs of 0.03 to 0.25 mg/L, resulting in the classification of one isolate as intermediate based on its MIC of 0.25 mg/L. However, a single aa exchange Glu434Asp in GrlB was observed in the QRDRs. Six isolates with doxycycline MICs of 0.25 mg/L were classified as intermediate.

The results of the quality control strain *S. aureus* ATCC^®^ 29213 were always within the respective QC ranges [38,39].

### 2.5. Investigation of the Reduced Susceptibility to Oxacillin

Agar disk diffusion for penicillin resulted in zone diameters of 8–18 mm, which classified the isolates as resistant [38,39]. The ST1 isolates displayed remarkably larger zone diameters (16–18 mm) than the ST1660 isolates (8–12 mm) (Table 3). A similar situation was observed for oxacillin, ampicillin, ampicillin-sulbactam and amoxicillin-clavulanic acid, whereas the amoxicillin zone diameters were comparable for the isolates of both STs and measured up to 8 mm (Table 3). Comparing the results for ampicillin, and ampicillin-sulbactam as well as for amoxicillin and amoxicillin-clavulanic acid, increased zone diameters (of ≥5 mm) were detected for the β-lactamase inhibitor containing compounds. The results obtained with the quality control strains *S. aureus* ATCC^®^ 25923 and *Escherichia coli* ATCC^®^ 35218 were always within the respective QC ranges [38,39].

Regarding the reduced susceptibility to oxacillin, the Fem (factors essential for methicillin resistance) proteins [42] were further investigated, including the aa sequences of FemA, FemB, FemX, FemC and FemD. Compared to the β-lactam susceptible *S. aureus* reference strain ATCC^®^ 25923 (acc. no. CP009361.1), the following situation was observed. Within the FemA protein sequence, all isolates displayed the aa alteration Glu234Asp, while the ST1 isolates had an additional difference, Tyr195Phe. Moreover, the ST1 isolates displayed the aa alteration Arg199Ser in the FemB sequence. Within the FemX protein sequence, all isolates had an Asn18His alteration. While all ST1 isolates exhibited only one additional aa exchange (Ile51Val), the ST1660 isolates showed two additional exchanges (Asn155Thr and Thr262Lys) in the FemX protein sequence. There were no differences regarding the aa sequences of FemC and FemD.

All isolates had aa differences in the catabolite control protein A (CcpA), a global transcriptional regulator, shown to be associated with oxacillin resistance in staphylococci [43,44]. The following differences were observed: Lys171Glu and Ser207Gly, and the ST1 isolates showed an additional Ala197Glu exchange.

All ST1660 isolates had two aa differences (Ile456Val and Asp561Glu) in the phosphodiesterase GdpP, for which is known, that mutations can lead to elevated oxacillin MICs [45,46]. The position Ile456Val is located in the so-called desert hedgehog (DHH) domain [47,48].

Being the primary targets of β-lactam antibiotics, the penicillin binding proteins (PBPs) [42] were also comparatively investigated. The PBP aa sequences of the analyzed isolates were compared to the respective ones of the reference strain *S. aureus* ATCC^®^ 25923 (acc. no. CP009361.1). Differences could be detected in all investigated PBP aa sequences. All ST1 isolates showed two aa exchanges in PBP1 (Asp118Asn, Val617Met), three in PBP3 (Gly167Arg, Lys504Arg, Asp563Glu) and one in PBP4 (Thr189Ser). The ST1660 isolates displayed a single variation in PBP2 (Val102Met), four differences in PBP3 (Gly167Glu, Ala330Ser, Lys504Arg, Asp563Glu) and one in PBP4 (Glu398Ala). Moreover, all isolates had an elongation of eleven aa at position 717 in the PBP2 aa sequence.

Furthermore, the aa sequences of the additional proteins that have been shown to be involved in methicillin resistance, like the efflux pump regulator MgrA [49] and the multiple peptide resistance factor MprF [50] involved in cell wall synthesis, showed no differences to the respective aa sequences of *S. aureus* ATCC^®^ 25923.

### 2.6. Overexpression and Induction of blaZ

**Figure 3:**
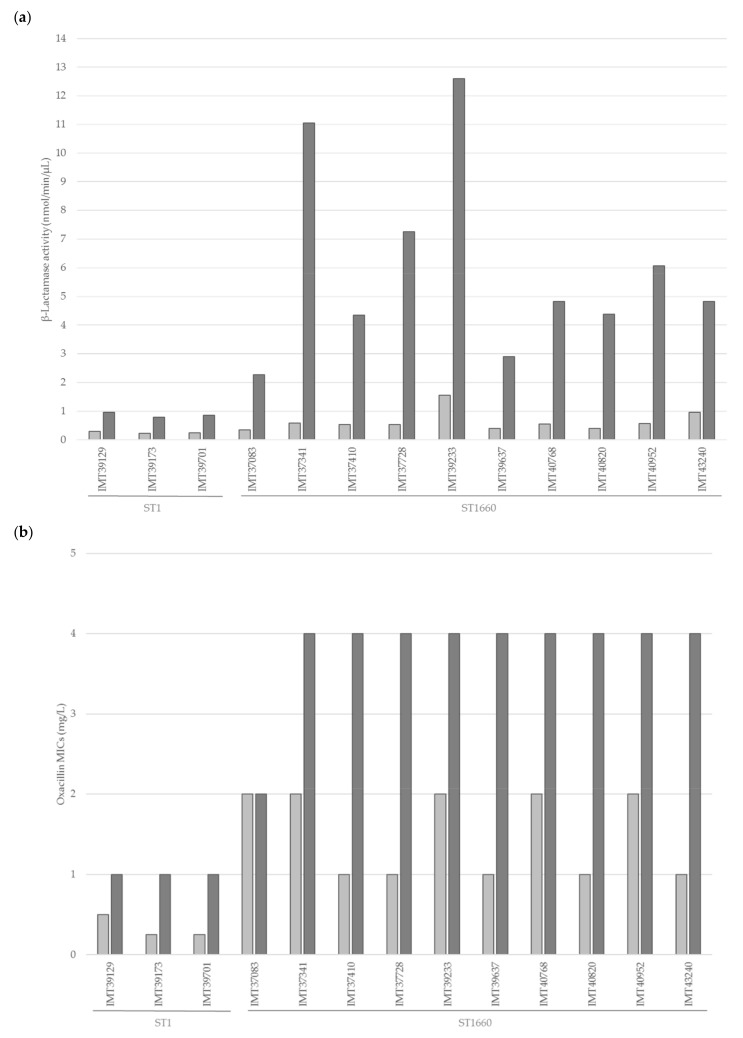
(**a**) Mean β-lactamase activity (nmol/min/µL) obtained by testing in duplicate and (**b**) oxacillin MIC values of selected isolates before and after induction. Light grey = before induction, dark grey = after induction.

The representative isolates (all three ST1 and ten ST1660) were subjected to β-lactamase activity testing using a nitrocefin assay. The induction with ampicillin revealed a 5.05–19.10-fold increase of β-lactamase production for the ST1660 isolates, while the ST1 isolates showed only a moderate increase (3.17–3.40-fold) (Figure 3a). The Mann-Whitney-U-Test revealed a significant difference (*U* = 0.0; *p* = 0.007) between the ST1 and the ST1660 isolates. Susceptibility testing after induction resulted, for all but one isolate, in oxacillin MICs that were one to two dilution steps higher than the corresponding values of the uninduced isolates (Figure 3b). The isolate IMT37083 (ST1660) showed no change in its oxacillin MIC of 2 mg/L. For the remaining nine ST1660 isolates tested, oxacillin MICs increased to 4 mg/L, thus they were classified as resistant. Even though the ST1 isolates showed increased oxacillin MICs of 1 mg/L, they were still classified as susceptible according to the CLSI standards [38,39].

**Figure 4:**
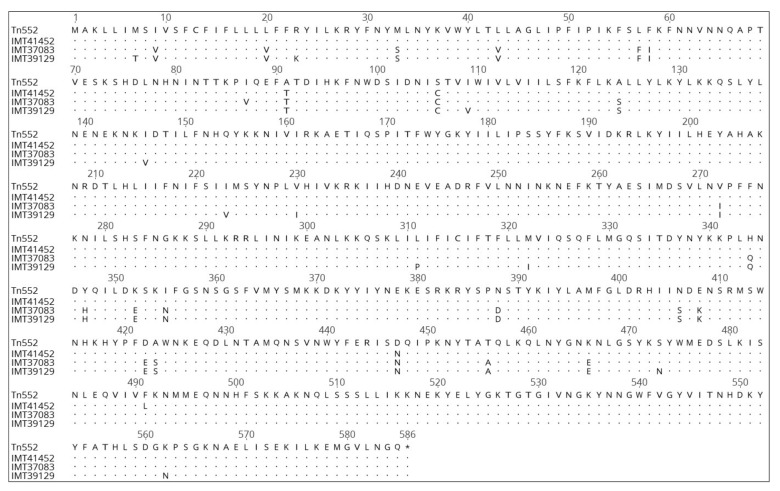
Comparison of the aa sequences of BlaR1 of IMT41452, which represents the majority of the ST1660 isolates, except the differing ST1660 isolate IMT37083, and IMT39129, which represents the ST1 isolates. The transposon Tn*552* of *S. aureus* NCTC 9789 (acc. no. X52734.1) served as a reference. The dots indicate the identity of aa, while letters indicate aa exchanges.

The investigation of the β-lactamase operon containing genes encoding the proteins BlaZ, BlaR1 and BlaI revealed the differences between the sequence types. The aa sequences were compared to the originally described sequence of *S. aureus* transposon Tn*552* (acc. no. X52734.1) [51]. For the repressor BlaI, only one aa alteration, Gly21Asp, was observed which was present in all ST1 isolates and the ST1660 isolate IMT37083. The BlaZ proteins of the ST1 isolates had seven aa exchanges (Ser22Pro, Val86Ile, Glu145Gly, Tyr220Cys, Val9Ala, Ala112Glu and Pro217Ser). The aforementioned ST1660 isolate IMT37083 shared the latter three aa exchanges (Val9Ala, Ala112Glu and Pro217Ser) with the three ST1 isolates and harbored the additional aa exchange Lys169Arg. In the BlaR1 sensor protein, all ST1 isolates showed 32 aa exchanges, with 19 of them also occurring in the aforementioned ST1660 isolate IMT37083 (Figure 4). The remaining ST1660 isolates had four aa exchanges in BlaR1 (Ala91Thr, Ser106Cys, Asp447Asn, and Phe491Leu).

### 2.7. Susceptibility to Biocides

Comparative biocide susceptibility testing via broth macrodilution revealed MICs of 0.00006–0.0005% for benzalkonium chloride (BAC), 0.125–0.5% for glutardialdehyde (GLU) and 0.00006–0.00025% for chlorhexidine (CHX). The broth microdilution results were 0.000125–0.0005% (BAC), 0.25–0.5% (GLU) and 0.000125–0.00025% (CHX) (Table 4). Overall, the results vary between three to four dilution steps in broth macrodilution and between two to three dilution steps in broth microdilution. The biocide MIC values of the ST1 and ST1660 isolates did not show major differences. Only a slight difference could be seen regarding the CHX MIC values. The *S. aureus* reference strain ATCC^®^ 6538 was tested for comparative reasons and showed comparable results to previous studies [37].

## 3. Discussion

Between 2015 and 2017, BORSA isolates caused infections in horses of a German equine clinic. These isolates were initially noticed as they showed elevated MICs for oxacillin using VITEK2. However, these MICs were classified as susceptible.

An analysis of whole genome sequencing (WGS) data revealed two lineages being associated with the conspicuous oxacillin phenotypes, ST1-t127 and ST1660-t3043, -t2484 and -t549. ST1 is attributed to CC1, which is known to be a livestock-associated putative pathogen, causing zoonotic infections [18]. The isolates with similar characteristics to our isolates (ST1-t127, ST1660-t549 and ST1660-t3043) have been previously described as MRSA and MSSA in equine samples [6,11,21,25,26] and have also been detected in samples obtained from humans in different European and non-European countries [28,29,30,31,32,33]. In addition, MSSA ST1660 with other *spa* types, t2484 for *S. aureus* from a horse in Germany [52] and t3043 for an isolate obtained from a donkey in Tunisia [53] were described. The isolates attributed to ST1-t127 belong to the three most prevalent lineages of MRSA in the Italian pig industry [54,55] and were also detected in cattle in Italy and China, [56,57] and wild boars in Germany [58].

The two clusters obtained by cgMLST were in accordance with the multi locus sequence typing (MLST) and could further differentiate the isolates. Interestingly, the four ST1660-t3043 isolates obtained in 2015 cluster very closely together, showing 0-2 allelic differences only, while the isolates obtained in other years were more distantly related (Figure 2). The correlation of the cgMLST and the *spa* types revealed that the 14 isolates with *spa* type t3043 could be assigned into 11 allelic profiles by cgMLST. However, it should be noted that one allelic profile was shared by two isolates with different *spa* types, namely t549 and t3043. A former study on the relatedness of *S. aureus* outbreak isolates using the SeqSphere+ cgMLST approach revealed that genomes with 0 to 8 allelic differences should be considered as related, while those with 9 to 29 allelic differences seemed to be possibly related, and those with 30 or more differences were rated as unrelated [59]. However, a lack of epidemiological metadata concerning possible relatedness of individual cases forbids further speculation here. Moreover, a recent study on equine MRSA obtained from horses directly at hospital admission revealed a very limited number of genomic differences for unrelated equine ST398 MRSA as well [13].

All isolates were negative for PVL and the toxic shock syndrome toxin 1, a T-cell activating superantigen (SAg). The ST1660 isolates carried further genes encoding SAgs, including enterotoxins and enterotoxin-like proteins, which are beyond others associated with *egc*. However, only non-*egc* encoded SAgs have been implicated in toxin-mediated diseases [60]. Thus, the role of *egc*-encoded SAgs in equine *S. aureus* requires further investigation. At present, there is an ongoing discussion about the impact of enterotoxins on colonization abilities of *S. aureus.* The ST1 isolates harbored the enterotoxin gene *seh*, which is usually attributed to CC1 and the corresponding protein is known for its binding affinity to human major histocompatibility complex class II [61]. The ST1 and ST1660 isolates were positive for the leukocidin genes *lukP/Q* located on a bacteriophage most similar to Saeq1 (acc. no. LT671578) [62]. Very recently, this phage was reported for MRSA-ST398 isolated from horses in the same area [13]. LukPQ preferentially kills equine neutrophils, but it also showed activity towards human neutrophils at high concentrations [62]. Moreover, an important immune-modulating factor, a variant of Scin, is also located on that phage. Previous research indicated a C3-inhibiting activity of *eq*SCIN in plasma of a much broader range of hosts, including horses, humans, and pigs [63]. According to Monecke et al., most isolates of CC1 harbor a β-hemolysin converting phage, which is supported by our findings [64].

Regarding the antimicrobial resistance properties, the ST1 isolates were resistant to penicillins, aminoglycosides, enrofloxacin, sulfamethoxazole/trimethoprim and tetracyclines and accordingly classified as multiresistant, based on their resistance to three or more classes of antimicrobial agents [65]. Similar resistance profiles were also detected among BORSA isolates of ST1 and ST1660 and MRSA CC398 from horses in equine clinics [13,21]. The ST1660 isolates were resistant to penicillins, and aminoglycosides. It should be mentioned, that except for penicillin, enrofloxacin and doxycycline, no equine-specific clinical breakpoints for the tested antimicrobial agents were available [38].

Regarding the reduced oxacillin susceptibility, the VITEK2 results were compared with the broth microdilution results. The broth microdilution results were generally lower than the results obtained by VITEK2. Moreover, higher MICs were determined for the ST1660 isolates compared to the ST1 isolates.

All isolates lacked the known *mec* genes, encoding an additional PBP and causing resistance to virtually all β-lactams, except specific anti-MRSA compounds [38]. Moreover, no gene with considerable homology to *mec* genes could be detected in the wholegenome sequences so far. Therefore, other potential causes were analyzed. An analysis of the PBPs, compared to the susceptible *S. aureus* ATCC^®^ 25923 (acc. no. CP009361.1) revealed aa alterations in PBP1, PBP2, PBP3 and PBP4, even though none of these differences were within the functional transglycosylase or transpeptidase domains [1,2,66,67,68]. Since Morroni et al. compared the PBPs of ceftobiprole-resistant MRSA isolates with the vancomycin-resistant *mecA*-carrying MRSA Mu50 [69], a comparison of our isolates with Mu50 (acc. no. NC_002758.2) was performed. Some of the mutations present in Mu50 compared to *S. aureus* ATCC^®^ 25923, were also present in our collection, including the eleven aa terminal elongation in PBP2, but also the aa differences Gly167Arg, Lys504Arg and Asp563Glu in PBP3, as well as Thr189Ser (ST1) in PBP4. Since Mu50 is an MRSA isolate with an alternative PBP2a, it cannot be stated whether these aa changes are involved in the generation of elevated oxacillin MICs.

The mutations in the *gdpP* gene, which encodes a phosphodiesterase that regulates gene expression, have also been described in association with borderline oxacillin resistance [45]. The GdpP protein has two functional domains, GGDEF and DHH. The GGDEF domain contains a diguanylate cyclase, conferring the capacity to synthesize the second nucleotide messenger cyclic di-GMP, and the DHH domain contains the phosphodiesterase characteristic catalytic DHH motif, mediating hydrolysis of cyclic di-AMP, which is, besides others, involved in cell wall homeostasis [47,48]. Only one aa difference of the ST1660 isolates (Ile456Val) is located in the DHH domain. The fact that one aa alteration is located in a functional domain might indicate a possible contribution to the reduced oxacillin susceptibility of the ST1660 isolates. These alterations were only present among the ST1660 isolates and, therefore, did not explain the BORSA phenotype of the ST1 isolates. However, the comparatively lower oxacillin MICs confirmed for the ST1 isolates versus the ST1660 isolates could possibly be in accordance with this finding. A study by Griffiths and O’Neil revealed that neither a mutation Asp418Ala within the DHH domain nor a truncation of GdpP to 370 aa, causing a deletion of this domain, contributed to oxacillin resistance [45]. Therefore, the aa changes in the GdpP protein reported here for ST1660 isolates might have little or no effect on the phenomenon of elevated oxacillin MICs in this study.

In the case of MRSA, some genes, e.g., those of the *fem* family, are important for methicillin resistance [70]. However, the respective Fem proteins were found in the WGSs of susceptible and resistant *S. aureus* isolates. In MRSA, the inactivation of these factors results in a Fem-specific reduced resistance to oxacillin in the corresponding strains, ranging from slightly decreased MICs to complete hypersusceptibility to β-lactam antibiotics [42,71]. Thus far, studies only showed a reduction of oxacillin resistance in usually resistant MRSA isolates, when these factors were altered or deleted [42,70,72]. Consequently, the involvement of the Fem alterations in the increase of oxacillin MICs is not likely.

In line with this, the deletion or inactivation of the carbon catabolite protein CcpA was only shown to increase the β-lactam susceptibility in *S. aureus*, including MRSA, *Staphylococcus epidermidis* as well as different streptococci [43,44,73,74]. Therefore, the detected aa differences did not seem to play a role in the reduced oxacillin susceptibility.

Among staphylococci, the resistance to penicillins is commonly mediated by the *blaZ* gene. This is in accordance with this study, since all isolates were classified as penicillin-resistant by broth microdilution and agar disk diffusion and carried the *blaZ* gene. As expected, the β-lactam compounds containing a β-lactamase inhibitor revealed larger zone diameters than the respective β-lactam compound alone. All isolates showed zone diameter differences of ≥5 mm for the combinations with a β-lactamase inhibitor, indicating the presence of an active β-lactamase. This is in accordance with the results from previous studies. Maalej and colleagues revealed the differences of at least 5 mm for oxacillin zone diameters with and without clavulanic acid [75].

Borderline oxacillin resistance can be caused by the overexpression of the β-lactamase gene *blaZ* [16]. β-Lactamase hyperproduction was evaluated as an underlying mechanism, since oxacillin is usually no target for β-lactamases. Using a nitrocefin assay for the selected isolates, inducible β-lactamase production was detected in all 13 isolates tested. Interestingly, the ST1660 isolates showed a higher degree of induction, which might explain the higher oxacillin MICs (Figure 3). Subsequent susceptibility testing after induction with ampicillin, revealed that all isolates but one showed higher oxacillin MICs than without induction. While all ST1 isolates were still classified as susceptible, nine of ten tested ST1660 isolates reached oxacillin MICs of 4 mg/L and were classified as resistant. While all other ST1660 isolates displayed almost no differences to the originally described BlaZ-BlaR1-BlaI proteins of transposon Tn*552* of *S. aureus* strain NCTC 9789 (acc. no. X52734.1), ST1660 isolate IMT37083 shared 19 of the 32 aa differences within the respective proteins with the ST1 isolates. Since BlaR1 is the sensor protein for extracellular β-lactam antibiotics [51,76,77,78], it is possible, that these aa changes result in a comparatively lower expression of *blaZ* in ST1 isolates and IMT37083 in the presence of β-lactam antibiotics. The observed increase of the oxacillin MICs after induction of all but one isolate (IMT37083) tested points towards borderline oxacillin resistance due to β-lactamase hyperproduction [16,75].

Biocide susceptibility testing was performed for BAC, GLU, and CHX using broth macro- and microdilution as an additional characterization of the isolates [37,79]. In the equine clinic, only BAC is used, as a compound of a floor disinfectant (7.6 g BAC per 100 g disinfectant). The MICs for GLU and CHX of the isolates in this study were below the standard concentrations used and did not differ remarkably from the MICs of the *S. aureus* reference strain ATCC^®^ 6538. BAC is often used as an additive and not as a single antibacterial agent. Regarding BAC, the highest MIC of the isolates corresponds to the lowest used concentration of this agent.

In conclusion, in a two-year period, two closely related lineages of *S. aureus*, causing infections in an equine clinic, were identified. These isolates were attributed to sequence types/clonal complexes (including MRSA) that are commonly isolated from equine samples, but have also a zoonotic potential [21,22,23,24,25,26]. Here, borderline oxacillin resistance seems to be associated with the hyperproduction of the β-lactamase BlaZ. The detection and correct classification of isolates expressing the BORSA phenotype is of major importance since the effectiveness of β-lactams is limited and therapy failure might occur when these isolates cause infections.

## 4. Materials and Methods

### 4.1. Background Information and Bacterial Isolates

During the years 2015-2017, routine diagnostics identified 19 *S. aureus* isolates with elevated MICs for oxacillin via VITEK2. These isolates originated from 17 equine patients of a veterinary clinic (Table 5). One sample was from an injured horse and one from respiratory disease. All other samples were from surgical site infections. Most of the cases (eleven samples from nine patients) were from orthopedic procedures. Four samples were from colic patients and two from surgeries of the genito-urinary tract. All surgical patients were treated with a combination of amoxicillin and gentamicin, either as single shot therapy before surgery or up to six days after surgery. The injured patient and four with surgical site infections were additionally treated with the combination of sulfamethoxazole/trimethoprim from three up to 13 days. One of these patients had to undergo a second surgery where a single shot dose of amikacin was injected.

### 4.2. Characterization of the Isolates

The DNA extraction for WGS was performed using the QIAamp^®^ DNA Mini Kit (QIAGEN, Hilden, Germany) with some adaptations for staphylococci. Before starting the official protocol, the cells were mixed with 25 µL lysostaphin solution (0.1 mg/mL) and incubated for 25 min at 37 °C. Then, 75 µL TE buffer and 25 µL proteinase K (0.1 mg/L) were added and the cells were incubated for 25 min at 37 °C. Then, 75 µL PBS and 2 µL RNAse A (2 µg/µL) were added and slightly mixed. After this, the protocol for the kit was followed starting with the addition of AL buffer. The libraries for WGS were prepared using the Nextera XT library preparation kit (Illumina Inc., San Diego, CA, USA) according to the manufacturer’s instructions. The 2 × 300 bp paired-end sequencing in 30-fold multiplexes was performed on the Illumina MiSeq platform. The genome sequences were *de novo* assembled using Newbler (Roche, Basel, Switzerland) and SPAdes v3.12.0 [80]. The nucleotide sequences were analyzed with Geneious v11.1.4 (Biomatters Ltd., Auckland, NewZealand) and annotated with the subsystem technology server (RAST) [81] and Prokka [82] which were compared with BLAST (National Center for Biotechnology Information, Rockville Pike, USA) [83] results. Further investigations were performed by using ResFinder [84] of the Center for Genomic Epidemiology (http://www.genomicepidemiology.org/). The virulence factors were identified by using VFanalyzer of VFDB (http://www.mgc.ac.cn/cgi-bin/VFs/v5/main.cgi?func=VFanalyzer) and checked using Geneious v11.1.4. The associated mobile genetic elements including pathogenicity islands and phages were determined using Geneious v11.1.4 software. The STs were derived from the pubmlst database (https://pubmlst.org) and *spa* types were deduced using the Ridom Spa Server (http://www.spa.ridom.de).

Whole genome sequences were analyzed for the proteins known to play a role in oxacillin resistance: GdpP, FemA, FemB, FemC (GlnR), FemD (GlmM), MgrA, CcpA and MprF as well as the penicillin binding proteins (PBP) 1, 2, 3 and 4. The sequences of these proteins were compared with *S. aureus* ATCC^®^ 25923, an oxacillin-susceptible reference strain (acc. no. CP009361.1). The *blaZ-blaI-blaR1* operon was compared to the respective region of the originally described transposon Tn*552* (acc. no. X52734.1) [51]. The analyses were carried out with Geneious v11.1.4.

### 4.3. Phylogenetic Analysis

The molecular epidemiology was investigated using the previously generated WGSs as FASTA files for the *S. aureus* core genome multilocus sequence typing (cgMLST) approach. For this, the software SeqSphere+ version 6.0.2 (Ridom GmbH, Münster, Germany) was used [85,86]. To illustrate the clonal relationship between the different isolates, a minimum-spanning tree was built based on a distance matrix of the core genome allelic profiles including 1744 of 1861 possible target genes, using the “pairwise ignoring missing values” option of the software.

### 4.4. Antimicrobial Susceptibility Testing

The routine diagnostics performed antimicrobial susceptibility testing via VITEK2 according to the manufacturer’s instructions. Additional susceptibility testing to 31 antimicrobial agents was performed by broth microdilution according to the CLSI recommendations [38,39] using sensititre^TM^ microtiter plates. *S. aureus* ATCC^®^ 29213 was used for quality control. The antimicrobial susceptibility testing by broth microdilution was repeated for the β-lactam antibiotics using selected isolates (all three ST1 and ten ST1660 isolates), after the induction with ampicillin [0.25 µg/mL (ST1) or 32 µg/mL (ST1660) ampicillin (Roth^®^, Karlsruhe, Germany)], to investigate the effects of induced β-lactamase production. The inoculum for the induction testing was prepared using the growth method, where the isolates were incubated with the respective amount of ampicillin for 4 h in cation-adjusted Mueller-Hinton broth (CAMHB) and then, the bacterial suspension was adjusted to McFarland 0.5. Additionally, the susceptibility to kanamycin (Roth^®^, Karlsruhe, Germany) was tested via broth macrodilution [38,39].

### 4.5. Investigation of β-Lactamase-Production

Agar disk diffusion [39] was performed using BBL™ Sensi Discs for penicillin (10 IU), oxacillin (1 µg), ampicillin (10 µg) and amoxicillin/clavulanic acid (20/10 µg) and Oxoid™ discs for amoxicillin (10 µg) and ampicillin/sulbactam (10/10 µg) on Mueller-Hinton agar (MHA) plates. *S. aureus* ATCC^®^ 25923 and *E. coli* ATCC^®^ 35218 were used for quality control purposes, according to CLSI standard [39]. The results of agar disk diffusion for β-lactam antibiotics with and without a β-lactamase inhibitor were comparably investigated.

Using a nitrocefin-based β-Lactamase Activity Assay Kit (Sigma-Aldrich^®^, Munich, Germany), the β-lactamase activity of all ST1 isolates (*n* = 3) and selected ST1660 isolates (*n* = 10) were quantitatively investigated. Therefore, the isolates were cultured overnight in brain-heart-infusion (BHI, Oxoid, Wesel, Germany) at 37 °C. The next day, 5 mL BHI with and without 0.25 µg/mL (ST1) or 32 µg/mL (ST1660) ampicillin (Roth^®^, Karlsruhe, Germany) were inoculated with 200 µL of the overnight cultures and incubated for 4 h at 37 °C. Following the manufacturer’s instructions, the reaction was prepared in duplicate with a sample volume of 20 µL per isolate and the absorbance was measured at a wavelength of 490 nm, every 60 s for one hour. The standard curves were evaluated for every microtiter plate and β-lactamase activity was calculated, according to the manufacturer’s instructions. 

### 4.6. Biocide Susceptibility Testing

Comparative biocide susceptibility testing was performed using a broth macrodilution method [37] and a broth microdilution method, which has been developed in this research group [79]. *S. aureus* ATCC^®^ 6538 was tested for comparative reasons and the results were compared to those of a previous interlaboratory trial [37]. The optical densities were adjusted according to German Veterinary Medical Society (DVG) standards for biocide efficacy testing [87]. For broth microdilution, twofold dilution series were prepared in 100 µL per well on a 96 well plate. The biocide solutions were prepared in deionized water and the inoculum was prepared in tryptic soy broth (TSB). The inoculum was added to a final testing volume of 200 µL and the results were read after incubation for 24 h at 37 °C. The MIC was defined as the first well concentration without visible growth. The tested biocides were BAC (Roth^®^, Karlsruhe, Germany), GLU (Roth^®^, Karlsruhe, Germany) and CHX (Sigma^®^, Munich, Germany). The test ranges were prepared in twofold dilution series; 0.000008–0.004% for BAC, 0.008–4% for GLU and 0.00001–0.0005% for CHX.

### 4.7. Statistical Analysis

The statistical analysis was performed using IBM^®^SPSS^®^ Statistics Version 25. To compare the β-lactamase inducibility of the isolates, non-parametric Mann-Whitney-U-test was performed.

### 4.8. WGS Submitted to GenBank

This Whole Genome Shotgun project has been deposited at GenBank under the accession numbers VSYP00000000 (IMT41899), VSYQ00000000 (IMT43240), VSYR00000000 (IMT43231), VSYS00000000 (IMT43228), VSYT00000000 (IMT41468), VSYU00000000 (IMT41452), VSYV00000000 (IMT40952), VSYW00000000 (IMT40820), VSYX00000000 (IMT40768), VSYY00000000 (IMT39841), VSYZ00000000 (IMT39233), VSZA00000000 (IMT37728) VSZB00000000 (IMT37410), VSZC00000000 (IMT37341), VSZD00000000 (IMT37083), VSZE00000000 (IMT39637), VSZF00000000 (IMT39701), VSZG00000000 (IMT39173) and VSZH00000000 (IMT39129).

## Figures and Tables

**Table 1:** Virulence genes of the 19 equine *S. aureus* isolates.

ST	Isolate	*hlb*	*fnbA*	*fnbB*	*ica*	*sei*	*selm*	*seln*	*selo*	*ϕent2*	*selq*	*seh*	*lukD/E*	*lukP/Q*
**ST1**	IMT39129	-	+	+	+	-	-	-	-	-	-	+	+	+
**ST1**	IMT39173	-	+	+	+	-	-	-	-	-	-	+	+	+
**ST1**	IMT39701	-	+	+	+	-	-	-	-	-	-	+	+	+
**ST1660**	IMT39637	+	+	+	+	+	+	+	+	+	+	-	+	+
**ST1660**	IMT37083	+	+	+	+	+	+	+	+	+	+	-	+	+
**ST1660**	IMT37341	+	-	+	+	+	+	+	+	+	+	-	+	+
**ST1660**	IMT37410	+	+	+	+	+	+	+	+	+	+	-	+	+
**ST1660**	IMT37728	+	+	+	+	-	+	+	+	+	+	-	+	+
**ST1660**	IMT39233	+	+	+	+	+	+	+	-	+	+	-	+	+
**ST1660**	IMT39841	+	-	+	+	-	+	+	+	-	+	-	+	+
**ST1660**	IMT40768	+	+	+	+	+	+	+	+	+	+	-	+	+
**ST1660**	IMT40820	+	+	+	+	+	+	+	+	+	+	-	+	+
**ST1660**	IMT40952	+	+	+	+	+	+	+	+	+	+	-	+	+
**ST1660**	IMT41452	+	+	+	+	+	+	+	+	+	+	-	+	+
**ST1660**	IMT41468	+	+	+	+	-	+	+	+	+	+	-	+	+
**ST1660**	IMT43228	+	+	+	+	+	+	+	+	+	+	-	+	+
**ST1660**	IMT43231	+	+	+	+	+	+	+	+	+	+	-	+	+
**ST1660**	IMT43240	+	+	+	+	-	+	+	+	+	+	-	+	+
**ST1660**	IMT41899	+	-	-	+	-	+	+	+	+	+	-	+	+

+ = gene present, - = gene absent or deleted

**Table 2:** Distribution of minimal inhibitory concentrations (MIC) values of the 19 different equine *S. aureus* isolates.

	No. of Isolates with MIC (mg/L)																Susceptible		Intermediate		Resistant	
Antimicrobial Agent(s)	0.015	0.03	0.06	0.12	0.25	0.5	1	2	4	8	16	32	64	128	256	512	no.	%	no.	%	no.	%
Oxacillin	-	-	-	-	2	1	9	7	-	-	-						19	100	-	-	-	-
Penicillin	-	-	-	-	-	-	-	3	-	-	-	-	16				-	-	-	-	19	100
Ampicillin		-	-	-	-	-	1	2	-	-	-	-	1	15								
Amoxicillin/clavulanic acid ^a^		-	-	-	3	-	9	7	-	-	-	-	-	-								
Imipenem	12	7	-	-	-	-	-	-	-	-	-	-	-									
Ceftiofur		-	-	-	-	1	11	6	1	-	-	-	-	-								
Cefquinome	-	-	-	-	-	8	11	-	-	-	-	-	-									
Cefalothin			-	-	3	16	-	-	-	-	-	-	-	-	-							
Cefotaxime	-	-	-	-	-	-	-	11	8	-	-	-	-									
Cefoperazone			-	-	-	-	-	3	8	8	-	-	-	-	-							
Erythromycin	-	-	-	-	10	8	1	-	-	-	-	-	-				18	94.7	1	5.3	-	-
Tylosin tartrate			1	-	-	-	11	7	-	-	-	-	-	-	-							
Tulathromycin			-	-	-	-	-	-	3	12	4	-	-									
Tilmicosin			-	-	-	-	12	7	-	-	-	-	-	-	-							
Clindamycin		-	-	15	4	-	-	-	-	-	-	-	-	-			19	100	-	-	-	-
Pirlimycin		-	-	-	-	13	6	-	-	-	-	-	-	-								
Tiamulin		-	-	-	-	4	15	-	-	-	-	-	-	-								
Ciprofloxacin	-	-	-	9	6	1	2	1	-	-	-	-					18	94.7	1	5.3	-	-
Enrofloxacin	-	1	4	10	1	2	1	-	-	-	-	-					15	78.9	1	5.3	3	15.8
Marbofloxacin	-	-	-	-	14	2	3	-	-	-	-	-										
Nalidixic acid			-	-	-	-	-	-	-	1	15	-	3	-								
Gentamicin				-	-	-	-	-	-	-	-	7	11	1	-	-	-	-	-	-	19	100
Kanamycin						-	-	-	-	-	-	-	5	14								
Streptomycin					-	-	-	-	-	14	5	-	-	-	-	-						
Neomycin				-	-	11	5	-	-	2	1	-	-	-								
Tetracycline				-	16	-	-	-	-	-	-	1	2	-	-	-	16	84.2	-	-	3	15.8
Doxycycline			-	10	6	-	-	-	1	2	-	-	-	-	-		10	52.6	6	31.6	3	15.8
Sulfamethoxazole/trimethoprim^a^	-	-	-	1	-	7	8	-	3	-	-	-	-				16	84.2	-	-	3	15.8
Florfenicol				-	-	-	-	-	18	1	-	-	-	-	-	-						
Linezolid		-	-	-	-	-	2	14	3	-	-	-	-	-			19	100	-	-	-	-
Vancomycin	-	-	-	-	-	8	11	-	-	-	-	-	-				19	100	-	-	-	-
Quinupristin/dalfopristin	-	-	-	-	10	9	-	-	-	-	-	-	-				19	100	-	-	-	-

Grey shading indicates concentrations not included in the test panel. Isolates with growth throughout the panel have MIC values equal to or larger than the highest concentration tested and are, therefore, displayed in the next higher concentration with grey shading; classification as susceptible, intermediate or resistant, according to CLSI [38,39] is indicated by black vertical bars; species-specific clinical breakpoints for horses were applied for penicillin, enrofloxacin and doxycycline, the remaining breakpoints were adopted from human medicine [38,39]. ^a^ amoxicillin and trimethoprim MIC values were used for the combinations amoxicillin/clavulanic acid (2:1) and sulfamethoxazole/trimethoprim (19:1), respectively.

**Table 3:** Zone diameter values of the 19 *S. aureus* isolates.

ST ^a^	Isolate	Zone Diameter (mm) ^b,c^					
		PEN (10 U)	OXA (1 µg)	AMP (10 µg)	SAM (10/10 µg)	AMX (20/10 µg)	AMC (10 µg)
ST1	IMT39129	**18**	20	18	26	7	30
ST1	IMT39173	**18**	19	18	22	8	30
ST1	IMT39701	**16**	19	17	26	8	30
ST1660	IMT39637	**10**	8	9	18	no zone	22
ST1660	IMT37083	**12**	14	12	19	7	22
ST1660	IMT37341	**10**	12	10	16	no zone	20
ST1660	IMT37410	**10**	12	10	18	no zone	20
ST1660	IMT37728	**10**	12	10	18	no zone	20
ST1660	IMT39233	**10**	14	10	18	no zone	22
ST1660	IMT39841	**8**	14	10	16	no zone	20
ST1660	IMT40768	**9**	14	9	16	no zone	21
ST1660	IMT40820	**10**	14	10	17	no zone	20
ST1660	IMT40952	**11**	14	10	18	no zone	21
ST1660	IMT41452	**9**	14	10	18	no zone	20
ST1660	IMT41468	**10**	15	10	17	7	20
ST1660	IMT43228	**10**	14	11	18	no zone	21
ST1660	IMT43231	**10**	14	8	17	7	22
ST1660	IMT43240	**12**	15	12	20	7	24
ST1660	IMT41899	**11**	14	11	19	7	22

^a^ ST = sequence type; ^b^ PEN = penicillin, OXA = oxacillin, AMP = ampicillin, SAM = ampicillin-sulbactam, AMX = amoxicillin, AMC = amoxicillin-clavulanic acid ^c^ Bold numbers indicate resistance according to CLSI [38].

**Table 4:** MIC distribution obtained with broth micro- and macrodilution.

Method ^a^ and ST	BAC ^b^ (MIC in %)				GLU (MIC in %)			CHX (MIC in %)		
	0.00006	0.000125	0.00025	0.0005	0.125	0.25	0.5	0.00006	0.000125	0.00025
Micro ST1				3	1	2				3
Macro ST1			3			2	1			3
Micro ST1660		1	11	4		13	3		9	7
Macro ST1660	1	1	5	9	3	12	1	4	12	

^a^ Micro = broth microdilution, Macro = broth macrodilution ^b^ BAC = benzalkonium chloride, GLU = glutardialdehyde, CHX = chlorhexidine. Numbers indicate the numbers of isolates showing the respective MIC.

**Table 5:** Background data for the *S. aureus* isolates.

Isolate	Date	History	Surgery	Sample Material	Age	Sex
IMT39129 ^a^	9 April 2016	arthritis	yes	synovial fluid	5 years	gelding
IMT39173 ^a^	11 April 2016	arthritis	yes	tissue sample	5 years	gelding
IMT39701	12 July 2016	fracture	yes	wound sample	<1 year	stallion
IMT39637	22 June 2016	trauma	no	wound sample	n.k.	gelding
IMT37083	13 July 2015	fracture	yes	wound sample	14 years	mare
IMT37341	24 August 2015	colic surgery	yes	abscess	8 years	stallion
IMT37410	11 September 2015	castration	yes	wound sample	1 year	gelding
IMT37728	5 October 2015	colic surgery	yes	wound sample	11 years	gelding
IMT39233	25 April 2016	rupture of the urinary bladder	yes	wound sample	2.5 weeks	stallion
IMT39841	11 August 2016	wound healing disorder	yes	wound sample	14 years	gelding
IMT40768	7 December 2016	fracture	yes	wound sample	4 years	mare
IMT40820	28 December 2016	colic surgery	yes	wound sample	1 year	mare
IMT40952	17 January 2017	colic surgery	yes	TBS	8 years	gelding
IMT41452	27 January 2017	wound healing disorder	yes	wound sample	15 years	gelding
IMT41468	2 February 2017	sinusitis	yes	wound sample	5 years	gelding
IMT43228 ^b^	16 June 2017	fracture	yes	wound sample	7 years	mare
IMT43231 ^b^	19 June 2017	fracture	yes	wound sample	7 years	mare
IMT43240	22 June 2017	colic surgery	yes	wound sample	6 years	gelding
IMT41899	15 February 2017	fracture	yes	wound sample	14 years	mare

Isolates originating from the same patients are indicated with the same superscript letters. TBS = tracheobronchial secretion, n.k. = not known.
